# Lifestyle E-Coaching for Physical Activity Level Improvement: Short-Term and Long-Term Effectivity in Low Socioeconomic Status Groups

**DOI:** 10.3390/ijerph16224427

**Published:** 2019-11-12

**Authors:** Hanne Spelt, Thomas Tsiampalis, Pania Karnaki, Matina Kouvari, Dina Zota, Athena Linos, Joyce Westerink

**Affiliations:** 1 Philips Research, 5656 AE Eindhoven, The Netherlands; joyce.westerink@philips.com or; 2 Eindhoven University of Technology, 5612 AZ Eindhoven, The Netherlands; 3 The Institute of Preventive Medicine, Environmental and Occupational Health Prolepsis, 151 25 Athens, Greece; t.tsiampalis@prolepsis.gr (T.T.); p.karnaki@prolepsis.gr (P.K.); m.kouvari@prolepsis.gr (M.K.); d.zota@prolepsis.gr (D.Z.); a.linos@prolepsis.gr (A.L.)

**Keywords:** lifestyle e-coaching, physical activity improvement, socioeconomic status

## Abstract

E-coaching applications can improve people’s lifestyles; however, their impact on people from a lower socioeconomic status (low SES) is unknown. This study investigated the effectiveness of a lifestyle e-coaching application in encouraging people facing low SES disadvantages to engage in a more active lifestyle over a course of 19 weeks. In this bicountry study, 95 people with low activity level (GR: 50, NL: 45) used a mobile application linked to a wearable activity tracker. At the start and after 6 and 19 weeks, self-reported physical activity levels, attitudes, and intention towards increasing activity levels, perceived behavioral control, and wellbeing were measured. Results indicated that participants using the lifestyle e-coaching application reported significantly more often an increase in activity levels than a parallel control group. Additionally, the people using the application also more often reported increased levels of wellbeing and perceived behavioral control. Therefore, lifestyle e-coaching applications could be a cost-effective solution for promoting healthier lifestyles in low-SES populations.

## 1. Introduction

Worldwide, 1 in 4 adults does not meet the physical activity recommendations set by the WHO [1]. Lifestyle e-coaching applications are known to have the potential to be successful in changing people’s behaviors, including physical activity levels [2,3]. Presently, various systems and devices for lifestyle improvement are on the market, e.g., the FitBit and Samsung Gear wearables, plus their accompanying apps. They guide people towards desired lifestyles through measurement of relevant parameters (e.g., activity level, food intake), and by means of personalized coaching messages. Their accompanying apps can function as mobile health applications, as, for example, physical activity is a prerequisite for good overall health. E-coaching applications, such as those used through a smartphone, have shown promising results in increasing physical activity among adults, senior citizens, children, and adolescents [2,4,5,6,7]. Nevertheless, scholars conclude that more research is needed on incorporating elements of behavioral change theories and conducting randomized controlled trials with the apps [6,8,9,10,11]. Improving physical activity levels can also improve wellbeing, although there is inconclusive evidence concerning e-coaching applications and their effect on wellbeing [12,13]. However, socioeconomic factors might influence the positive effects of lifestyle e-coaching on health and wellbeing, with researchers emphasizing the need to incorporate different algorithms that take into consideration different population characteristics and needs [10].

At the moment, the main way to obtain such a lifestyle improvement device is by buying it for a considerable price, making it a solution solely for people with the means to afford them. However, unhealthy behaviors are more common among people with a lower socioeconomic status (SES), among others as a result of coping with the stress of their status [14,15]. SES can influence important determinants for healthy behavior, i.e., self-regulation [14,16] and executive functioning [17]. Early childhood stress by environmental stimuli such as poverty is likely to cause deficits in these processes [14]. Therefore, lifestyle e-coaching might be extra beneficial for people from low SES. However, little research on e-coaching in lower SES groups has been conducted so far. This paper therefore investigated whether lifestyle e-coaching applications are also able to change behavior in groups with a lower SES. We start with a description of the positive effects of lifestyle e-coaching, followed by a discussion of their specific use by people in lower SES groups, and complete these findings with our research rationale.

### 1.1. E-Coaching for Behavior Change

E-coaching uses technology to support people in making healthy behavioral choices, often by including monitoring and feedback of said behavior [18]. These interventions can use several validated techniques to change behavior [19]. However, it is not easy to change human behaviors as they depend on underlying psychological motivations, such as attitudes or intentions, as well as capability and opportunity [20,21]. The COM-B model states that to change behavior, people need to be capable, motivated, able, and have an opportunity to change their behavior [20]. Many e-coaching systems try to influence these factors and thus behavior by indicating opportunities for change, as well as the underlying psychological factors, by providing information, personalized feedback, social comparison, and persuasive messages. According to the theory of planned behavior [22,23], attitude towards the specific behavior, subjective norm, and perceived behavioral control shape the intention towards, and thus the actual behavior. An important underlying assumption in persuasive technology is that people aim for cognitive consistency [24]; if attitudes and/or behaviors are not aligned, a person will become motivated to reduce this inconsistency. Thus, to achieve sustained behavior change, e-coaching systems need to change the ideas about the behavior in order to change that behavior. Once a change in behavior has been achieved, it is important to maintain this improvement for a longer duration, such that the new behavior patterns are not just temporary but a persistent change in lifestyle. Further, this aspect is usually reflected in the person’s attitude and intentions.

Research indeed shows that lifestyle e-coaching applications have the potential to successfully change people’s lifestyles [2,3]. However, these studies were mostly targeted at (motivated) people in the general public. This may have biased the results, as these people are already aware of the need for change and might have the motivation to change their lifestyles indicated by their participation in the study. Potentially, they have more knowledge and resources to do so. The effects might be different for different groups. For many people, changing lifestyle, such as increasing the level of physical activity, is not a goal in itself. Those who like being physically active are usually not the ones who need an e-coaching application to reach the advised physical activity levels. For many other people, becoming more physically active is a means to an end, for instance, to gain better mental and physical health, and through that to reach a higher level of wellbeing. This effect on wellbeing might especially be relevant for lower SES groups. Of course, it is clear that there are many other factors impacting wellbeing as well. Social, economic, and environmental factors shape health and wellbeing both directly and indirectly via health behaviors. The local context (e.g., country, income, access to medical care) can even be expected to moderate the effects of e-coaching on wellbeing or health. Research indicates that the effects of SES level on health are both direct, e.g., context, and indirect, e.g., unhealthy behaviors that form in this context [14,15]. Lifestyle e-coaching applications might be able to improve health for people with a lower SES via that indirect pathway. However, social, economic, and environmental factors can be expected to moderate the effects on lifestyle e-coaching on health and wellbeing.

### 1.2. Effectiveness of Lifestyle E-Coaching among Groups with a Lower Socioeconomic Status

Presently, it is unclear whether e-coaching would indeed help people in lower SES groups to improve their behaviour. Several reasons can be imagined why this approach would be less effective, one of them being a lack of commitment to lifestyle improvement if the wearable is freely available: In the Transtheoretical Model [25], an important step in the process of behavior change is “contemplation”, and persons who are simply given a device might have one less reason to contemplate their motivation for behavior change sufficiently, which could have a negative impact on their final results. A second reason e-coaching in people with a lower SES might be less effective is that people with a lower SES might have to spend more of their (mental) resources to making ends meet in their everyday life and managing their limited economic resources, for example, in paying rent, or feeding a family. As a result, this group has little room for such interventions. Research indeed indicates that people with a lower SES are difficult to reach with personalized lifestyle interventions [14].

Several reasons can be imagined why lifestyle e-coaching would be equally effective for different SES groups. Self-regulatory capacity and executive functioning differ between SES groups due to differences in their primary focus, childhood development, and the load on their mental resources [14]. As a result, these groups might have lower capabilities to change behavior [20]. Indeed self-regulation and executive functioning are important for sustained healthy behaviors, but e-coaching applications might reduce the load on these resources for this particular behavior change. Additionally, e-coaching devices try to change behaviors by influencing underlying motivational factors [20], such as attitudes, intentions, or perceived behavioral control [22]. Not all those factors are subject to socioeconomic status. This might suggest that e-coaching applications could be equally effective for different SES groups.

### 1.3. Research Rationale

While it is becoming clear that health consciousness might be lower in groups with a lower SES [26], at the moment, little information is available on the impact of e-coaching for these groups. Research shows that lifestyle e-coaching applications can change health-related behavior of the general public [2,3]. To the best of the authors’ knowledge, only a few studies are available that investigate whether the beneficial effects can also be expected for people in lower SES groups. A recent meta-analysis of electronic and mobile health interventions in developing countries showed positive impacts on physical activity and healthy nutrition-related behaviors, but results are not conclusive [27].

In order to investigate this possibility, we conducted this study on the effect of e-coaching applications aiming to increase physical activity levels. A certain amount of moderate physical activity per week (150–300 min) is generally advised to foster good health, but it is known that this threshold is not reached by a large percentage of the population [28]. The level of physical activity can easily be measured with wearable technology [29], which allows personalized e-coaching for physical activity enhancement. On the other hand, even only wearing such a device can already influence the level of physical activity of the user [30]. This might hamper the acquisition of a reliable baseline physical activity level. Therefore, in academic investigations, retrospective questionnaires are often used for assessing physical activity level, for instance, (the short version of) the International Physical Activity Questionnaire (S-IPAQ) [31,32]. In the S-IPAQ, the user is asked to reflect on their physical activity levels before using the wearable tracker.

To achieve persistent behavior change, e-coaching applications focus—apart from changing the physical activity level of the user itself—also on changing the psychological aspects that form these behaviors, such as attitudes and intentions towards them [23]. Even if the actual behavior did not change, a lasting change in factors such as attitude and intention can be considered an important step forward. In that respect, their levels over time are relevant for sustained behavior change. Attitudes and intentions can be assessed via questionnaires related to various models of behavior change, for instance, the theory of planned behavior [22].

If e-coaching applications can reduce unhealthy behaviors in lower SES groups, this is potentially advantageous to their health. However, it might only partially decrease health inequality, since the link between lower SES and worse health is both indirect via unhealthy (stress-coping) behaviors and direct via exposure to social, economic, and environmental stressors [14,15]. Especially people from a lower socioeconomic status (low SES) living in underprivileged areas are provided fewer opportunities for safe, affordable, and appropriate programs or services to become more physically active [33], even while physical inactivity is a determining factor in many chronic conditions [4,10,34]. The contexts of lower-SES people can have a direct influence on health and wellbeing [14] by limiting opportunities for change [20]. For example, a perceived lack of neighborhood safety reduces physical activity level in children [35]. Context determinants can be country, income, household, (type of) employment and education, as well as actual SES level [36], and these can also be expected to moderate the effect of e-coaching on wellbeing or physical activity level. 

In conclusion, the primary objective of the current study was to investigate whether a lifestyle e-coaching application can be effective in increasing physical activity (as primary outcome) in groups with a lower socioeconomic status (SES), and whether such an increase in physical activity level (if present at all) is sustained after prolonged use of the lifestyle e-coaching application. The related hypothesis is that after 6 weeks of use of a lifestyle e-coaching application, the subjective physical activity level of the participants has increased significantly in comparison to that of a control group without lifestyle e-coaching, and that after a prolonged use of 3 further months, this significant difference persists. The secondary objective was to investigate whether within the subgroup with a lower SES, the activity level improvement (if present at all) depends on the actual SES level.

## 2. Materials and Methods 

The methodology followed was based on the CONSORT statement for nonpharmacologic treatment interventions [37] in terms of deploying an age, gender, and educational status-matched control group as well as random assignment of participants to study groups (control and experimental). This study received ethics approval from dedicated ethical boards on both study sites. Participation was voluntary and based on informed consent. All data were stored in de-identified format using personal participant codes. For privacy reasons, only the participants could access the detailed data generated by the lifestyle e-coaching application.

### 2.1. Trial Design

The present study was a two-site (Athens in Greece, Eindhoven in the Netherlands), two-arm, parallel-group, randomized controlled trial (following the study of Wijsman et al. [2]), which proceeded in three phases over 19 weeks. Only the participants in the experimental group were given access to the lifestyle e-coaching system.

### 2.2. Participants

Participants were recruited using a recruitment agency per study site. Participants were selected to have a socioeconomic ISEI score lower than 145 (according to the International Socioeconomic Index (ISEI) described by Ganzeboom et al. [36]), an age between 18 and 65 years, and an estimated level of physical activity of less than 210 min of light activity per week (slightly higher than advised by Marshall et al. [29]). For practical reasons, they needed to be in possession of an iOS or Android smartphone (versions 9.0 and 5.0, respectively) and willing to install the mobile application and give informed consent. Exclusion criteria included pregnancy, a medical condition that required them to abstain from moderate physical activity, or if they were already logging their physical activity levels. Participants received reimbursement for their participation.

### 2.3. Intervention

The e-coaching application included an activity tracker, i.e., Samsung Gear Fit2 Pro, connected to a mobile application, i.e., Samsung S Health [38]. The e-coaching application can support lifestyle changes in various domains, e.g., weight, consumption, and sleep. Our study focused on daily active minutes. In the mobile application, participants set a target of at least 30 active minutes per day, which participant could increase if desired. The activity tracker can track behaviors by measuring geolocation, heart rate via photo plethysmography, and physical activity using accelerometry. Consequently, the activity tracker provides the mobile application with the relevant data for monitoring and coaching. The mobile application reports on these measurements and provides motivational messages based on activity insights. Motivational messages relate to the set target, i.e., ‘*be active: do not stay behind! Only 9 min until you reach today’s goal*’, or pop-up during inactivity, i.e., ‘*inactive time 50 min; time to get on your feet*’. Summaries include active minutes stratified in walking or cycling, number of steps, time, distance, and burned calories per activity, day or week. Coaching messages and summaries of behavior also appear on the activity tracker. The system allows users to support and compete against each other in an online community with monthly step-challenges. There was no intervention for the control group participants.

### 2.4. Questionnaires

Social economic status was assessed by means of the International Socioeconomic Index (ISEI) [36]. Additionally, demographic questions were asked, including age, gender, and family income. Subjective physical activity levels were measured using the short version of the International Physical Activity Questionnaire (S-IPAQ) [31], which measures different intensity levels of physical activity to estimate total physical activity in MET-min/week (metabolic equivalent of task) and sedentary behavior. The S-IPAQ is validated for the general population and young adults in Greece [39] and the Netherlands [31]. Mental wellbeing was measured with the short version of the Warwick–Edinburgh Mental Wellbeing Scale (WEMWBS) [40], which is validated for use in individuals aged 13 years old and older. Underlying motivations of behavior were assessed with a dedicated questionnaire based on the theory of planned behavior (TPB) [23] by measuring perceived behavioral control, the attitude towards and intention of behavior change. Table A1 presents the items of each subscale of the TPB questionnaire. Reliability of the S-IPAQ, WEMWBS, and TPB questionnaires was checked by calculating Cronbach’s alpha per country at baseline (see Table A2). 

Additionally, a composed user experience questionnaire measured usage and experiences with the e-coaching application in the experimental group. For the control group, a similar questionnaire inquired about the potential use of (other) activity tracking devices or mobile applications. All questionnaires were delivered in the local language.

### 2.5. Sample Size

The study of Wijsman et al. [2] describes how a significant effect was found of a lifestyle e-coaching application supporting users in increasing their physical activity levels after a number of weeks. As measured through objective measurements of physical activity (primary outcome), an effect size of d = 0.6 was found. Similar effect sizes have been found for mobile phone intervention studies targeting weight loss [3,41,42]. Assuming a similar effect size for our study, in which we also expected an increase in physical activity level (as primary outcome), we arrived at a sample size in each country of *n* = 45 per arm for a power of 0.80 (so total *n* = 4 × 45 = 180). To answer the primary objective, data collected in Greece and the Netherlands will be combined. This will increase the power to 0.95, even after correcting for an estimated dropout rate of 20%.

### 2.6. Procedure

Randomization of the participants in the two experimental groups was conducted by means of random number generation stratified for age, gender, and educational status in both countries. The experimental period spanned across 4.5 months (19 weeks). All participants were asked to report online on their physical activity levels, mental wellbeing, and underlying physical activity motivations at the beginning of the experiment (intake), after 6 weeks, and after 3 more months. Additionally, demographic information was gathered at the beginning and user experience in both follow-up questionnaires. 

Only the experimental participants were invited for an intake session, where they received the lifestyle e-coaching application and were helped with personalized installation of the system. They were instructed to wear the activity tracker (during the day) for a period of 6 weeks, set an activity goal of at least 30 min per day, and allow the mobile application to send push notifications. They were free to increase the physical activity goal or to add additional goals. At the end of the 6-week period, the experimental participants were informed that they could keep the application until the end of the experiment 3 months later and freely use it, although they were not obligated or required to do so. At the end of the experiment at 19 weeks, the experimental participants had the option of keeping the activity tracker instead of receiving the reimbursement. Experiment leaders were available the whole period to help with any problems with the lifestyle e-coaching application. 

### 2.7. Statistical Methods

Our primary objective questions whether an e-coaching application can increase physical activity, and thus wellbeing, levels (after prolonged usage). To answer this, our primary outcome measure was the likelihood of improving the physical activity level as measured by the IPAQ score in MET-minutes. The secondary outcome measures were the participants’ wellbeing, intention, attitude, and perceived behavioral control towards increasing physical activity levels.

Descriptive statistics are presented in absolute and relative frequencies for the categorical characteristics and in mean (standard deviation) or median (Interquartile Range, IQR) form for the continuous characteristics, separately for each intervention group as well as stratified by country. A Pearson chi squared test and a Fischer’s exact test were used for the categorical characteristics and a Mann–Whitney U test for the continuous characteristics in order to compare their distribution between the two intervention groups. A nonparametric-paired Wilcoxon test was used to compare the physical activity scores among the three time points within the same group.

A logistic regression analysis was employed to compare the average physical activity improvement of the two groups, as well as wellbeing, intention, attitude, and perceived behavioral control scores. As far as the primary outcome is concerned (physical activity), we ran three logistic regression models:

1. Model 1: Unadjusted;
2. Model 2: Adjusted for participants’ physical activity either at baseline or after 6 weeks;
3. Model 3: Adjusted for participants’ physical activity either at baseline or after 6 weeks, as well as demographic characteristics and initial motivational state.

With regard to our secondary measures, i.e., wellbeing, intention, attitude, and perceived behavioral control, only unadjusted results are presented. This article focuses on physical activity outcomes. Indicative results for the secondary measures are presented; more detailed analysis will follow in forthcoming papers.

Finally, our secondary objective questioned whether the improvement in physical activity (if present at all) depends on SES level. To answer our secondary objective, subgroup analysis according to participants’ physical activity level at baseline and their socioeconomic status was carried out, in order to investigate whether the effectiveness of the application is being significantly differentiated according to these characteristics. The Wilcoxon signed-rank test was used in order to determine the significance of the change in participants’ physical activity level. Statistical analysis was performed with the statistical software IBM SPSS v.25 (IBM Corp, Armonk, NY, USA) [43] and Stata v.13 (StataCrop, College Station, TX, USA) [44].

## 3. Results

**Figure 1:**
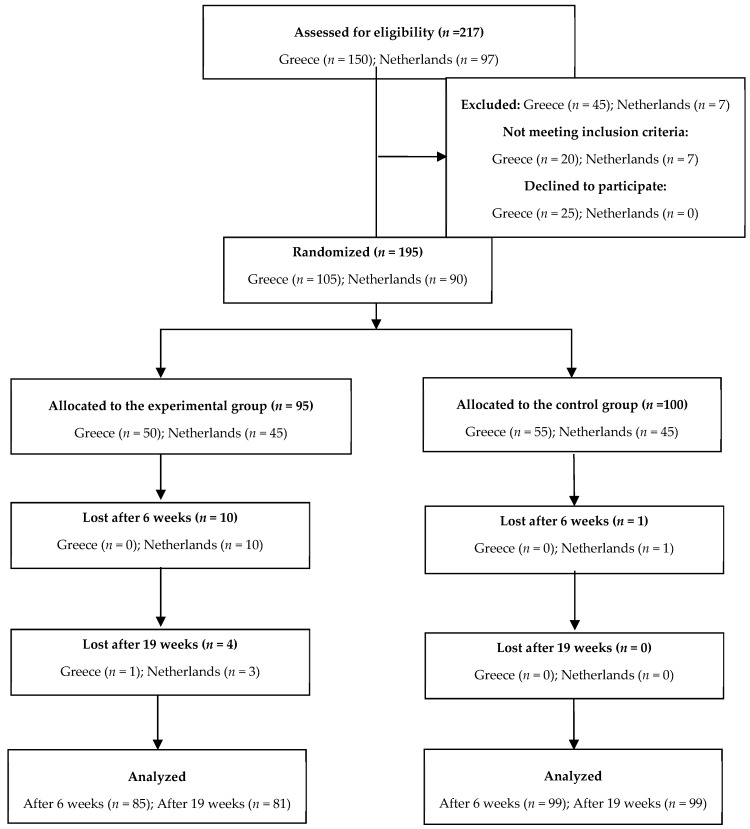
Flowchart of participants through study inclusion and participation.

As seen in the flow diagram (Figure 1), 217 people were assessed for eligibility (150 in Greece and 97 in the Netherlands), from which 195 (Greece: 105 and Netherlands: 90) met the inclusion criteria and were randomized in the two groups. More specifically, in Greece, 55 participants were allocated in the control group and 50 participants in the experimental, while in the Netherlands, 45 participants were allocated in each group. After 6 weeks, 11 participants were lost to follow-up and 4 additional participants after 19 weeks. Participants were lost in the follow-up when they did not fill in the second and/or the third questionnaire. Therefore, the final analysis was based on 184 participants for the 6-week period and on 180 participants for the 3-month period.

In Table 1, we present participants’ baseline characteristics for each group. Generally, participants had an average educational background, lived in a 3-member household, and had a lower socioeconomic status. Concerning participants’ physical activity level at baseline, the majority had low or moderate physical activity levels in both groups. In both countries, the two groups were not statistically significantly different for the baseline characteristics, with the exception of a significant difference in SES levels in the Netherlands, and a significant difference in baseline physical activity level in Greece. We controlled for baseline scores in our multivariate analysis, so as to adjust for the potential impact of these and other differences over groups.

**Figure 2:**
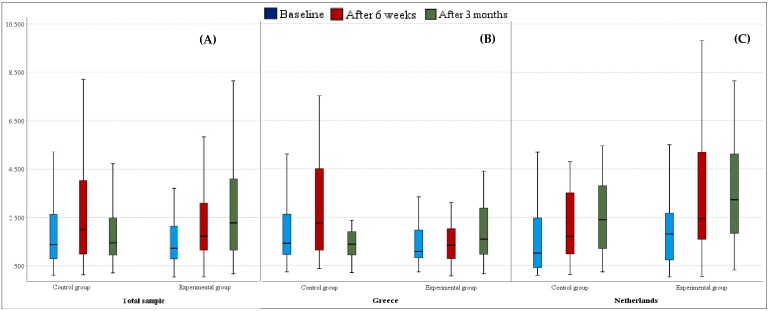
Boxplots representing participants’ physical activity levels as measured by the IPAQ score in MET-minutes at baseline, after 6 weeks, and after 19 weeks for each experimental group (**A**) in the total sample and separately (**B**) for Greece (*n* = 105) and (**C**) the Netherlands (*n* = 97). The middle line represents the median.

Physical activity levels of participants in the experimental group were significantly improved, both after 6 weeks (*p* < 0.001) and 19 weeks (*p* < 0.001) compared to baseline measurements (Table 2). Further, a significant difference was found between 6 and 19 weeks (*p* = 0.014), indicating the continued improvement of physical activity, while participants in the control group presented a significant improvement only after 6 weeks (*p* = 0.002). More importantly, the increase from baseline after 19 weeks tested significantly higher in the experimental compared to the control group (*p* = 0.002), as well as the increase between week 6 and week 19 (*p* = 0.007). In Figure 2, the development of the physical activity levels over time is presented, also for Greece and the Netherlands separately. 

Concerning the comparison between the two intervention groups (Table 3), a significantly higher percentage of participants in the experimental group succeeded in improving their physical activity level, either after 6 weeks (Experimental: 70.1%, Control: 65.0%) or after 19 weeks (Experimental: 80.0%, Control: 52.9%). Further, significantly more participants in the experimental group improved their physical activity between week 6 and week 19 than in the control group (Experimental: 66.2%, Control: 43.1%). In addition, also after adjusting for various participant’ characteristics (*Model 2* and *Model 3*), significant differences were found between the two groups with regard to their likelihood of improving their physical activity level. More specifically, participants in the experimental group had almost 4 times higher odds of improving their physical activity level after 19 weeks (OR = 3.74; 95% CI = 1.69–8.28; *p* = 0.001) and 3 times higher for improving it between week 6 and week 19 (OR = 2.65; 95% CI = 1.31–5.36; *p* = 0.007), when compared to the control group. With regard to the difference between the baseline and at 6 weeks, there was no significant difference between the two groups. 

We also examined the application’s effectiveness stratified by the participants’ physical activity level at baseline and by their socioeconomic status (Table 4). The results suggest that the lower the physical activity at baseline, the greater the improvement in physical activity level, either after 6 or after 19 weeks. More specifically, for low physically active participants, a statistically significant improvement was observed, both after 6 weeks (*p* < 0.001) and after 19 weeks (*p* = 0.001), while for the highly active participants, there was a significant change between 6 and 19 weeks (*p* = 0.012). With regard to the stratification based on the participants’ socioeconomic status, the physical activity level was found to improve significantly in both SES groups after 19 weeks (low SES: *p* < 0.001, very low SES: *p* = 0.008), while among the participants in the low-SES group, a significant improvement was noted after 6 weeks (*p* = 0.002), as well as between weeks 6 and 19 (*p* = 0.008). It should be noted though that the improvement observed in the low-SES group is not significantly different from the respective improvement in the very low-SES group (difference between the baseline and 6 weeks: *p* = 0.311; difference between the baseline and 19 weeks: *p* = 0.168; difference between 6 and 19 weeks: *p* = 0.236; results not shown).

We also examined whether the e-coaching application improved participants’ wellbeing, as well as their intention, attitude, and perceived behavioral control towards increasing physical activity levels (Table 5). More specifically, participants in the experimental group were found to have by 38% significantly higher odds of improving their wellbeing between weeks 6 and 19 compared to the control group (OR = 1.38; 95% C.I = 1.05–1.82; *p* = 0.026). There was no significant difference between the two intervention groups with regard to the improvement of intention or attitude, either after 6 or after 19 weeks. However, it was found that participants in the experimental group had by 36% significantly higher odds of improving their perceived behavioral control between weeks 6 and 19 when compared to the control group (OR = 1.36; 95% CI = 1.03–1.80; *p* = 0.035).

## 4. Discussion

This randomized controlled study investigated whether a lifestyle e-coaching application can be effective in increasing physical activity levels, wellbeing, and motivational state towards physical activity, e.g., intention, attitude, and perceived behavioral control, among low SES groups in Greece and the Netherlands. We found that indeed a lifestyle e-coaching application is capable of increasing physical activity levels of low-SES users, and moreover, of maintaining these increased physical activity levels. To our knowledge, this is the first randomized controlled trial to test an e-coaching application among people from a low SES and in two different countries.

### 4.1. E-Coaching Applications Can Increase Physical Activity Levels and Wellbeing among Low SES

As hypothesized, our findings showed moderate positive effects on physical activity levels after using the lifestyle e-coaching application for 6 weeks. These effects became stronger after a 3-month follow-up period. Since the increase in physical activity level as well as the likelihood of increasing it were significantly higher in the experimental group than in the control group, this increase in physical activity level can be attributed—at least in part—to the use of the lifestyle e-coaching application. These results are in line with a recent meta-analysis showing that the effectiveness of smartphone applications in increasing physical activity is evident at a 3-month term [4]. Interestingly, the increase in physical activity was more evident among people with lower physical activity levels as measured at baseline. Participants with low physical activity levels at the start showed an increase in physical activity levels in the post-intervention phase. Earlier studies have also shown promising results in increasing physical activity among previously sedentary adults using e-coaching apps [4,5,6,7,18], although they do not offer an explanation of their results. A possible explanation could be that for people with initially lower activity levels, it was easier to increase their capabilities, motivation, and indicate opportunities for improvement (based on the COM-B system by [20]). The initial inactivity might have resulted from unawareness of the benefits from an active lifestyle. As the lifestyle e-coaching app educates them and also points out opportunities for change, this could have increased (the awareness of) their physical behavior [20]. 

After 19 weeks of usage, participants that used the e-coaching application were more likely to report higher physical activity levels compared to participants who did not use the application. Other studies testing e-coaching applications have also shown a positive effect on participants who previously measured low on physical activity scores [45,46,47]. The fact that these effects only become significant after 19 weeks indicates on the one hand, that the impact of e-coaching applications is not immediate but needs several months to be visible, and, on the other hand, that a follow-up time considerably larger than 6 weeks is needed in a future study to assess the sustainability of these effects. It also confirms that behavior change is a long-term process and that an e-coaching system can provide appropriate long-term support. 

Wellbeing of participants using the application was also more likely to be improved between 6 and 19 weeks of usage compared to the control group. This finding signals that potentially e-coaching applications could have positive effects on the wellbeing of a population. The finding is, however, not in line with previous research. A study testing the effects of a mobile application on physical activity levels for stroke patients did not find any significant effect on psychological wellbeing [48]. Our study, however, concerns healthy people with no history of strokes, which could explain the different findings. Our findings urge future studies to examine the effects on wellbeing among people using wearables. 

In contrast to our expectations, the e-coaching applications did not increase motivational states in our study, i.e., increased attitudes and intentions towards increasing physical activity levels. Apparently, the lifestyle e-coaching application that we provided did not affect these aspects. The literature provides little evidence on how e-coaching applications affect intention and attitude scores, with findings being inconclusive and researchers indicating the need for further research [49,50]. Behavior change can result from changed attitudes and intentions [22], but this does not necessarily needs to be the case. Behaviors can also be changed by unconsciously providing opportunities for change, such as nudging. The specific mechanisms of changing behaviors using lifestyle e-coaching applications are interesting for future research. On the other hand, we did find that significantly more participants in the experimental than in the control group showed an increase in perceived behavioral control between 6 and 19 weeks: Apparently, the lifestyle e-coaching application helps users to feel more capable of managing their physical activity. A possible explanation could be that for people with initially lower activity levels, it was easier to increase their capabilities, motivation, and indicate opportunities for improvement (based on the COM-B system by [20]).

Concerning the secondary objective of this research, we observed that the long-term improvement of physical activity level attributed to the intervention was not associated with inter group SES variation; this could imply that the effectiveness of lifestyle e-coaching applications on physical activity levels is evident even among more underprivileged individuals. However, our study was not powered enough to allow further SES-based stratifications; hence, this outcome should be interpreted with caution.

### 4.2. Implications; Lifestyle E-Coaching Applications Can Be Low-Cost Solutions to Promote Healthy Lifestyles

The present findings suggest that the use of e-coaching applications may not only increase physical activity levels among people with a low SES but also have a positive effect on perceived wellbeing, especially for people with physically inactive lifestyles. The concurrent implementation of the study in two different countries Greece and Netherlands, characterized by different socioeconomic and cultural realities and the fact that similar results were observed, leads us to assume that the effect of lifestyle e-coaching applications is strong regardless of cultural backgrounds. Considering that other behavior modification opportunities may be less accessible for people of a low SES, e-coaching applications may be an easy and widely accessible solution to monitor and control detrimental lifestyle behaviors, such as physical inactivity. To address the issue of cost, inherently an issue with wearable technology used in e-coaching applications, it might be an option for local/national governments or insurance companies to provide them free of cost to those who cannot afford them. If lifestyle e-coaching applications can improve health-related behaviors in lower SES groups, this is potentially advantageous for their health. 

Research concerning e-coaching interventions to promote physical activity is scarce, even more so among people with a low SES. People with a low SES may not be able to take full advantage of e-Health and m-Health applications for various reasons, including lack of access to computers, mobile devices or Internet, social and cultural barriers in relation to digital literacy (e.g., Internet and smart device self-efficacy), or linguistic barriers. Indeed, overwhelming evidence exists as regards inequalities in accessing e-Health and m-Health applications [51]. However, considering that the number of users of smart devices such as smartphones is still increasing even within subgroups of individuals with a low socioeconomic status [52], the exploitation of their potential to promote health behaviors should be separately investigated in this target group.

### 4.3. Limitations and Future Research

To the best of our knowledge, this is one of the very few studies investigating the effect of an e-coaching application on the promotion of physical activity in a sample with people of a low socioeconomic status. There are some limitations to our study. Firstly, considering the limited sample size of this pilot, findings should be considered as exploratory evidence to build upon and guide a larger study trial. Secondly, data regarding physical activity levels were self-reported; hence, under- or over-reporting may exist, although validated scales were used. In addition, interventions targeting lifestyle behaviors may result in overestimated outcomes due to participants’ awareness of being observed. The intake session and the physical activity goals provided to the intervention group could have emphasized this observer effect. Lastly, the 3-month follow-up period does not allow generalizing observations to longer periods and to study long-term sustainability of these outcomes. 

## 5. Conclusions

A lifestyle e-coaching application tested among people of a low SES in Greece and the Netherlands was found to be successful in increasing physical activity levels after a 6-week intervention and the 19-week follow-up period. The impact appeared especially relevant among participants having low initial physical activity levels. Our experiment also showed a positive impact on wellbeing scores and perceived behavioral control. 

## Figures and Tables

**Table 1:** Baseline characteristics of the participants in both study sites (Greece, Netherlands).

	Greece (*n* = 105)			Netherlands (*n* = 97)		
	Experimental Group (*n* = 50)	Control Group (*n* = 55)	*p*-Value	Experimental Group (*n* = 45)	Control Group (*n* = 52)	*p*-Value
**Participants’ and household’s characteristics**						
**Gender** [*n* (%)]			0.391			0.770
Male	26 (52.0)	24 (43.6)		7 (15.6)	7 (13.5)	
Female	24 (48.0)	31 (56.4)		38 (84.4)	45 (86.5)	
**Age** [Mean (SD)]	39.4 (13.6)	40.2 (14.2)	0.769	42.9 (10.7)	42.0 (11.0)	0.674
**Level of education [*n* (%)]**			0.678			0.466
Low	1 (2.0)	1 (1.8)		7 (15.6)	4 (7.7)	
Middle	34 (68.0)	34 (68.0)		30 (66.7)	37 (71.2)	
High	15 (30.0)	15 (30.0)		8 (17.8)	11 (21.2)	
**Ethnic minority** [*n* (%)]	1 (2.0)	1 (1.8)	>0.999 ^a^	5 (9.6)	5 (11.1)	>0.999 ^a^
**No. of people in the family** [Median (IQR)]	3.0 (2.0–5.0)	3.0 (2.0–5.0)	0.981	3.0 (3.0–4.0)	3.0 (2.3–4.0)	0.948
**Number of children (below 18 years old) in the family** [Median (IQR)]	0.0 (0.0–1.3)	0.0 (0.0–1.0)	0.663	1.0 (1.0–2.5)	1.0 (1.0–2.0)	0.274
**SES score [Median (IQR)]**	35.3 (28.8–42.3)	39.0 (29.2–43.3)	0.081	41.0 (37.0–42.0)	38.0 (31.0–41.0)	**0.015**
**Outcome variables**						
**IPAQ score** [Median (IQR)]	1065.8 (722.0–1670.8)	1413.0 (906.0–2628.0)	**0.033**	1798.8 (669.0–2837.3)	1087.5 (432.8–2455.9)	0.159
**IPAQ score-categorized (%)**						
Low	20.4	8.2	0.065	41.0	31.8	0.393
Moderate	69.4	67.3		43.6	40.9	
High	10.2	24.5		15.4	27.3	
**WEMWBS score** [Median (IQR)]	28.0 (26.5–29.5)	28.0 (27.0–30.0)	0.812	27.0 (24.0–28.5)	27.5 (26.0–28.8)	0.322
**Intention score** [Median (IQR)]	6.0 (4.9–6.7)	6.0 (4.7–7.0)	0.729	6.0 (5.2–6.7)	6.3 (5.4–7.0)	0.078
**Attitude score** [Median (IQR)]	6.7 (6.2–7.0)	6.6 (5.8–7.0)	0.248	6.0 (5.4–6.4)	6.2 (5.8–6.6)	0.123
**Perceived Behavioral control score** [Median (IQR)]	6.3 (5.0–6.8)	6.0 (5.3–6.5)	0.583	6.0 (5.3–6.5)	6.3 (5.5–6.8)	0.224

**Note:***p*-values are based on Pearson chi squared test or the Fischer’s exact test when needed (^a^) for the categorical characteristics and on Mann–Whitney U test for the continuous characteristics and presented in bold if significant (*p* < 0.05). IQR = Interquartile range and is presented as the 25th–75th percentile of the characteristic’s distribution. SD = Standard Deviation. WEMWBS = Wellbeing score. SES = Socioeconomic status. Level of education: Low = any sort of education until high school, Middle = any sort of education until university, and High = any sort of education higher than the university. The categorization of the International Physical Activity Questionnaire (IPAQ) scores was based on the IPAQ scoring protocol [32].

**Table 2:** Physical activity level measured by the S-IPAQ in metabolic equivalent of task (MET)-minutes for each intervention group and its modification among the three time points.

Time Point	MET-Minutes/Week [Median (IQR)]		*p*-Value ^2^
	Experimental Group	Control Group	
Baseline	1198 (724–2124)	1345.5 (646–2468.6)	0.749
After 6 weeks	1662 (994–3066)	1777.5 (984–3942)	0.613
Difference between the baseline and week 6	475.5 (−137.0–1197)	319.5 (−215.8–1548.8)	0.688
***p*-Value ^1^**	**<0.001**	**0.002**	
After 19 weeks	2276 (1136–4086)	1440 (872.5–2478.2)	**0.022**
Difference between the baseline and week 19	876 (138–2536)	62.3 (−856.7–934)	**0.002**
***p*-Value ^1^**	**<0.001**	0.454	
Difference between week 6 and 19	330 (−334.8–1501.2)	−261.5 (−1240.5–593.6)	**0.007**
***p*-Value ^1^**	**0.014**	0.121	

**Note:**^1^ Tests the significance of the difference between the two time points based on the Wilcoxon test. ^2^ Tests the significance of the difference between the two intervention groups based on the Mann–Whitney U test. MET = Metabolic equivalent of task. IQR = Interquartile range presented as 25th–75th quantiles. *p*-values are presented in bold if significant (*p* < 0.05).

**Table 3:** Logistic regression results comparing the likelihood of improving physical activity level among the three time periods of study between the two groups.

Improvement of Physical Activity Level:	Percentage (%) of Participants Who Improved Their Physical Activity after Each Time Period				
	Experimental Group	Control Group	OR ^1^ (95% CI)	*p*-Value	After Adjusting for:
Between the baseline and week 6	70.1%	65.0%	1.13 (0.81, 1.56)	0.480	***Model 1***: Unadjusted
			1.19 (0.60, 2.35)	0.618	***Model 2***: IPAQ score at baseline
			1.10 (0.54, 2.23)	0.797	***Model 3***: IPAQ score at baseline, country, gender, age, SES, educational level, baseline intention score
Between the baseline and week 19	80.0%	52.9%	1.98 (1.27, 3.13)	**0.001**	***Model 1***: Unadjusted
			3.73 (1.72, 8.08)	**0.001**	***Model 2***: IPAQ score at baseline
			3.74 (1.69, 8.28)	**0.001**	***Model 3***: IPAQ score at baseline, country, gender, age, SES, educational level, baseline intention score
Between week 6 and week 19	66.2%	43.1%	1.61 (1.14, 2.27)	**0.005**	***Model 1***: Unadjusted
			2.63 (1.33, 5.19)	**0.005**	***Model 2***: IPAQ score after 6 weeks
			2.65 (1.31, 5.36)	**0.007**	***Model 3***: IPAQ score after 6 weeks, country, gender, age, SES, educational level, baseline intention score

**Note**: ^1^ OR = Odds ratio comparing the experimental versus the control group concerning the likelihood of improving the physical activity level after 6 and 19 weeks. CI = Confidence interval. *p*-value presented in bold if significant (*p* < 0.05). SES = Socioeconomic status. Level of education: Low = any sort of education until high school, Middle = any sort of education until university, and High = any sort of education higher than the university.

**Table 4:** Results of Wilcoxon signed-rank test comparing participants’ physical activity level expressed in MET-minutes at three time points, stratified by physical activity level at baseline (low–moderate–high) and socioeconomic status (very low–low).

	MET-Minutes/Week [Median (IQR)] Per Time Point			*p*-Values		
	*Baseline*	*After 6 Weeks*	*After 19 Weeks*	*Baseline—6 Weeks*	*Baseline—19 Weeks*	*6–19 Weeks*
**Stratified by participants’ physical activity level at baseline**						
**Low physical activity at baseline**						
*Experimental group*	438.0 (302.6–779.3)	1335.0 (862.5–2491.5)	1650.0 (991.5–3504.0)	**<0.001**	**0.001**	0.469
*Control group*	398.0 (292.0–676.0)	1440.0 (1071.0–3246.0)	2032.5 (942.0–3804.0)	**0.002**	**0.003**	0.776
**Moderate physical activity at baseline**						
*Experimental group*	1196.0 (951.0–1949.3)	1591.5 (896.6–2694.0)	1911.0 (1111.5–3363.8)	**0.007**	**<0.001**	0.171
*Control group*	1335.0 (885.0–1862.0)	1492.5 (942.0–3426.0)	1384.0 (823.3–2257.8)	**0.003**	0.535	0.135
**High physical activity at baseline**						
*Experimental group*	3093.0 (2462.5–5716.5)	2766.8 (1735.3–6127.5)	4510.5 (2122.8–8103.6)	0.836	0.121	**0.012**
*Control group*	4386.0 (2705.5–5138.6)	3846.0 (1680.0–5136.8)	1773.0 (1179.8–4216.9)	0.199	0.070	0.438
**Stratified by participants SES status**						
**Very low SES (below median)**						
*Experimental group*	1157.0 (809.8–2082.4)	1796.3 (896.6–3072.8)	2021.5 (1053.8–3825.8)	0.072	**0.008**	0.489
*Control group*	1422.0 (579.0–2826.0)	2133.0 (669.8–5193.8)	1435.5 (945.0–4489.9)	0.149	0.325	0.926
**Low SES (above median)**						
*Experimental group*	1224.0 (628.5–2142.0)	1662.0 (1230.3–2142.0)	2880.0 (1240.0–4992.8)	**0.002**	**<0.001**	**0.008**
*Control group*	1226.0 (756.0–2462.3)	1721.3 (1143.9–3435.0)	1440.0 (826.0–2388.0)	**0.005**	0.907	**0.049**

Note: SES = socioeconomic status. Physical activity categories were based on the IPAQ scoring protocol [32], while participants’ SES status was categorized according to whether their SES score was below or above the median SES score of the total sample (median SES score = 38.5 units). p-values are presented in bold if significant (*p* <0.05).

**Table 5:** Comparison between the two intervention groups, concerning the difference in their wellbeing, perceived behavioral control, intention, and attitude towards increasing physical activity levels at three time points during the study.

Unadjusted Logistic Regression Results Comparing Wellbeing, Intention, Attitude and Perceived Behavioral Control Improvement Levels over Time for Both Groups				
Percentage of Participants Who Improved Their:	Experimental Group (%)	Control Group (%)	OR (95% CI) ^1^	*p*-Value
**Wellbeing**				
Between baseline and week 6	38.3%	40.0%	0.97 (0.72, 1.29)	0.813
Between baseline and week 19	35.2%	26.2%	1.22 (0.91, 1.62)	0.199
Between week 6 and week 19	46.2%	29.8%	1.38 (1.05, 1.82)	**0.026**
**Intention**				
Between baseline and week 6	35.1%	25.6%	1.24 (0.93, 1.64)	0.159
Between baseline and week 19	35.2%	29.8%	1.12 (0.84, 1.50)	0.446
Between week 6 and week 19	30.8%	34.5%	0.92 (0.67, 1.26)	0.596
**Attitude**				
Between baseline and week 6	29.8%	27.8%	1.05 (0.77, 1.42)	0.764
Between baseline and week 19	34.1%	26.2%	1.19 (0.89, 1.59)	0.257
Between week 6 and week 19	41.8%	34.5%	1.16 (0.87, 1.54)	0.325
**Perceived behavioral control**				
Between baseline and week 6	34.0%	36.7%	0.95 (0.70, 1.28)	0.710
Between baseline and week 19	33.0%	26.2%	1.16 (0.87, 1.56)	0.327
Between week 6 and week 19	44.0%	28.6%	1.36 (1.03, 1.80)	**0.035**

Note: ^1^ OR = Odds ratio comparing the experimental and control group, CI = 95% confidence interval. *p*-value in bold if significant (*p* < 0.05).

## Appendix A

**Table A1:** Items for each subscale of the constructed questionnaire based on the Theory of Planned Behavior [23].

Scale	Items
Perceived behavioral control	For the next 6 weeks, achieving my daily goal is very likely for me I am confident that if I want, I can achieve my goal within the next 6 weeks Achieving my goal for the next 6 weeks is up to me I think I have full control of my goal for the next 6 weeks
Attitude towards behavior change	For me, to achieving my activity goal for the next 6 weeks is …. Responsible – Irresponsible Unpleasant – Pleasant Bad – Good Healthy – Unhealthy Detrimental – Beneficial
Intention to change behavior	I plan to achieve my daily goal for the next 6 weeks I will try to achieve my daily goal for the next 6 weeks I intend to achieve my daily goal for the next 6 weeks

**Note:** All items are answered on 7-point Likert scales.

**Table A2:** Reliability statistics of instruments at baseline for each country.

Instrument	α_Greece_	α_Netherlands_
Theory of planned behavior		
- Attitude towards behavior change	0.898	0.836
- Intention to change behavior	0.934	0.880
- Perceived behavioral control	0.896	0.785
Warwick-Edinburgh Mental Wellbeing Scale	0.887	0.821
Short International Physical Activity Questionnaire	0.854	0.862
